# Cyclic Peptidomimetic Lead Compounds to Reduce Neurotoxicity and Associated Oxidative Stress in Alzheimer’s Disease

**Published:** 2010-09

**Authors:** Antara Banerjee, Arpita Yadav

**Affiliations:** *Department of Chemistry, University Institute of Engineering and Technology, CSJM University, Kanpur, India*

**Keywords:** cyclic peptidomimetic, neurotoxicity, Alzheimer’s, zinc

## Abstract

Cyclic peptidomimetic compounds have been investigated utilizing ab initio Hartree Fock molecular orbital calculations. Prospective use of these ionophores as curative drugs for Alzheimer’s disease has been investigated by considering their metal toxicity removal efficiency. A (2,4,2,4) system that is, ((gly-et)-(ala-gly-gly-et))_2_ with CH_2_NH backbone is predicted to undergo low conformational reorganization in presence of Zn^2+^ ion with moderate electrostatic stabilization of ion. Conformation of this system and molecular weight render it suitable to be a lead compound for metal toxicity removal drugs required in AD.

## INTRODUCTION

Oxidative stress caused by various metal ions in human body leading to numerous associated problems like DNA damage, lipid peroxidation, depletion of protein sulfhydryls etc. is a rapidly proliferating arena for drug discovery. Several metals play an essential role in our body in controlling various metabolic and signalling pathways (1). Our body utilizes the redox chemistry of various metals including zinc, iron, copper, cobalt, manganese etc. However, when these metals ‘escape’ out of their normal course of action and designated site of actions they end up binding to arbitrary binding sites. These metals then end up reacting with nuclear proteins or DNA causing oxidative deterioration or binding to amyloid beta peptide (Aβ) causing unnatural changes in its shape leading to accumulation in the form of neuronal plaques. Examples of toxic effects of metals include hepatotoxicity, neurotoxicity and nephrotoxicity. Some toxic effects of various metals and oxidative stress exerted on our body due to their chemistry has been discussed at length in a recent review article (2).

Present study aims at designing preventive/curative type of drugs for Alzheimer’s disease. Current clinically used drugs mostly provide symptomatic relief (3). Alzheimer’s disease (AD) is also a result of oxidative stress on human brain caused by excess leakage of metal ions (4). AD is characterized by accumulation of amyloid beta peptide (Aβ) in brain in the form of senile plaques, in presence of zinc or copper metal ions enhancing its aggregation (5). Role of zinc in the etiology of AD is particularly fascinating as it plays the dual role of neuroprotection as well as neurodegeneration (6). It has been hypothesized that zinc binding to Aβ changes it’s conformation such that copper ions cannot bind to Aβ (7). This inhibits its aggregation and keeps it in solution form. However, some genetic or environmental factors may lead to abnormal Aβ metabolism that produces loads of zinc ions. This excess zinc instead of being neuroprotective triggers neuronal cell death. It has been experimentally observed that high concentrations of zinc force Aβ to precipitate over a wide range of pH (6).

Research in Alzheimer’s disease is focussed on designing preventive or curative type of drugs. Among preventive type β secretase peptide inhibitors have been extensively studied (8) and several compounds have shown activity in cellular assays. However, in terms of successful therapy peptidomimetic compounds with better pharmacokinetic properties are still awaited. Attempts at reparative compounds include anti aggregatory compounds and metal chelators like Co(III)-cyclen to keep Aβ in solution form (9-11). However, these chelators have undergone in vitro analysis and detailed toxicological investigations are needed to assess their human tolerability. Specificity and efficiency of metal chelator towards metal ion is of prime importance so that in all likelihood it removes only the toxic metal and not those needed by our body. For example, inorganic mercury has great affinity for sulfhydryl group. Therefore, drugs containing such functional groups like DMPS are used (12) for its removal. Natural compounds containing sulfhydryl groups like garlic (13) can also be used. α-lipoic acid can also be used to remove inorganic mercury but is not so efficient as it tends to loosely carry metal ion so as to leave it again inside body after picking up from site of action (14). This example has been cited to indicate complexities of metal detoxification.

We recently reported a lead compound of preventive type that would intercalate to Aβ keeping it in solution form and would also help remove metal toxicity (15). In our further quest for preventive or curative type of drugs for AD; we report here on mode of action of some cyclic peptidomimetic compounds that can remove metal ions from brain and thus reduce oxidative stress.

## METHODOLOGY

Ab initio Hartree Fock molecular orbital calculations (16) with complete geometry optimizations (17) have been performed with 6-31G (18) basis set. All calculations have been done using GAUSSIAN’03 software (19). GAUSSVIEW (20) has been used for all graphical purposes. Conformational reorganization required in compound to remove metal toxicity has been studied by geometry optimization in presence of ion. Energy loss in conformational reorganization and electrostatic gain in ion capture indicate the feasibility and suitability of compound to be used for metal toxicity removal. These values have been estimated as follows:

Conformational loss = E_reorgnized_ - E_original_ where both E correspond to geometry optimized stationary points.

E_original_ is in absence of ion and E_reorganized_ is in presence of ion. Electrostatic gain on capturing ion has been calculated by intermolecular interaction calculation of super-molecule type without BSSE estimation i.e.

Electrostatic gain= E_complex_- ( E_compound reorganized_ + E_ion_)

Effect of solvent has not been calculated because electrostatic gain on ion capture is much greater than the solvation energy of isolated ion leading to its desolvation and capture by drug molecule.

ADME properties of designed lead compounds have been predicted using QikProp module of Schrodinder (21) software.

## RESULTS AND DISCUSSION

We have investigated a series of cyclic peptidomimetic compounds with the aim of designing a lead compound for reducing neurotoxicity associated with Alzheimer’s. Following observations led to the choice of cyclic compounds.

- Cyclic compounds are less likely to ‘let go’ of the metal toxicity while removing it from body.
- Cyclic compounds with hydrophobic groups disposed outwards and having proper molecular weight can easily cross blood-brain-barrier.

**Figure 1:**
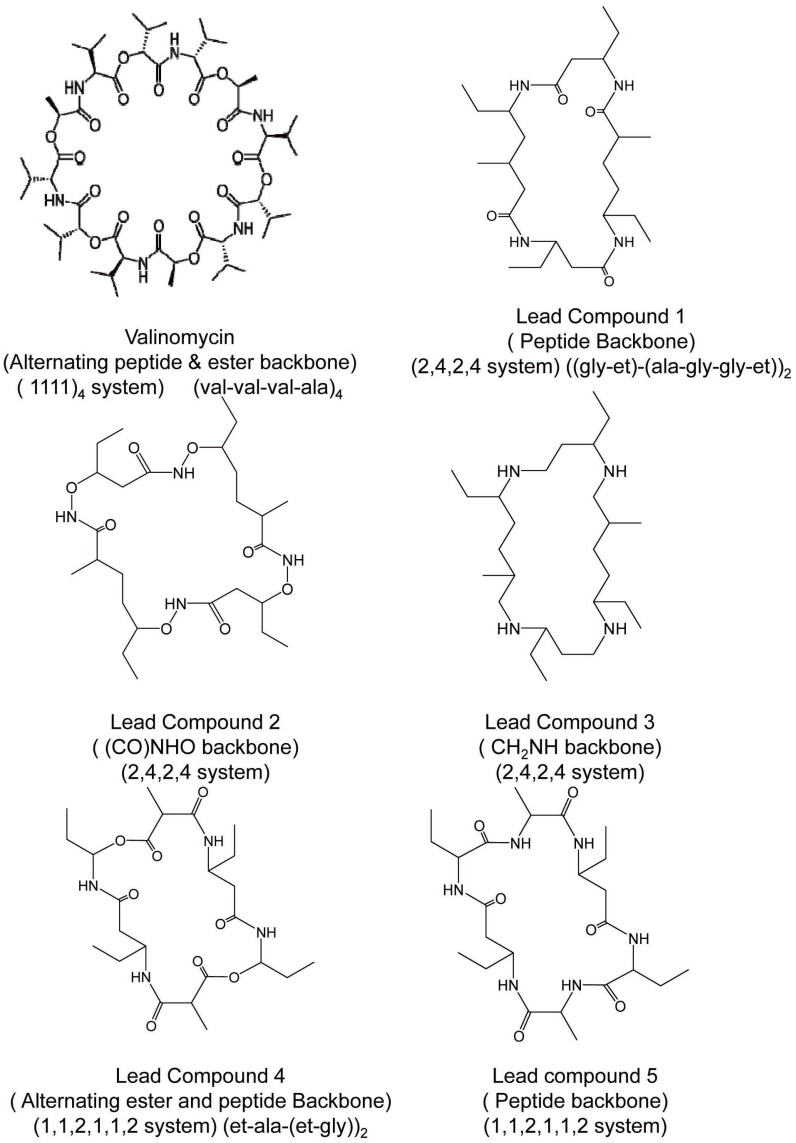
Valinomycin and designed cyclic ionophores.

**Figure 2:**
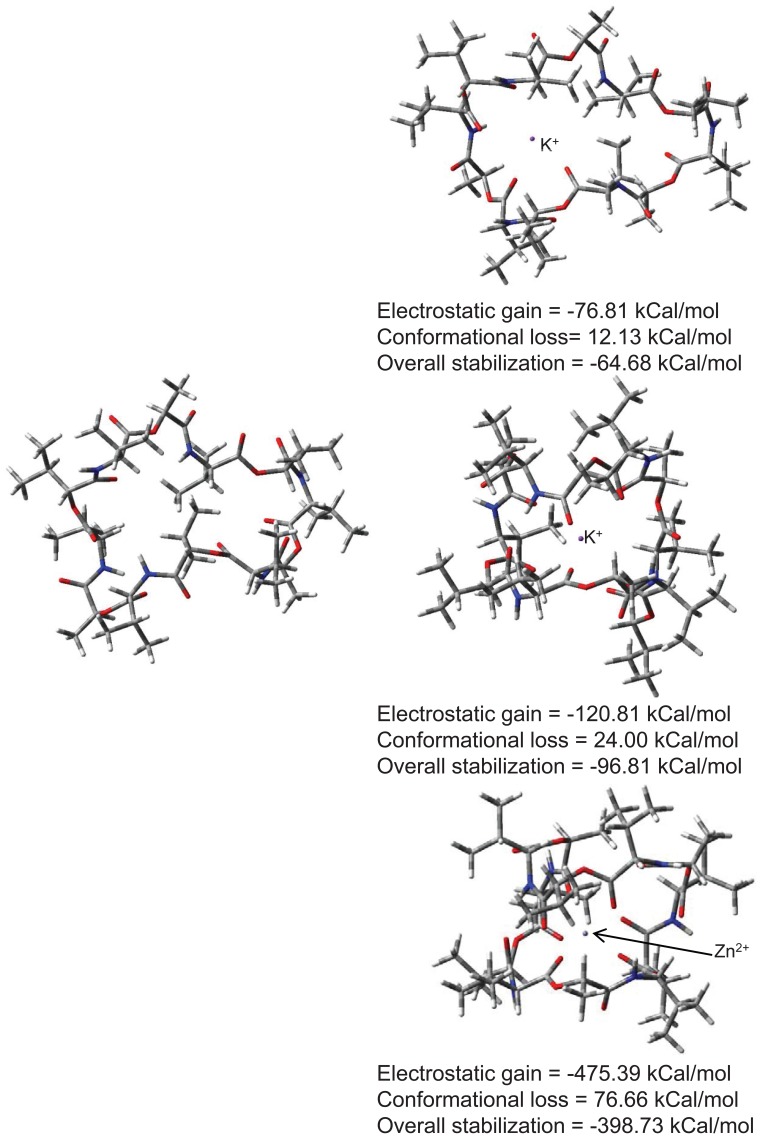
Conformational reorganization in Valinomycin to bind K^+^ or Zn^2+^ ions.

To proceed with designing in sequential fashion and to understand structural and electrostatic requirements for efficient metal toxicity removal we first studied mechanistic aspects of a known transport antibiotic valinomycin as our starting point. Valinomycin is an ionophore and acts by creating an influx of K^+^ ions in bacterial cells. Similar ionophores can be used to remove metal toxicity from human brain, keeping in mind that Valinomycin does not cross blood-brain-barrier (BBB) but can go through cell membranes. Possibilities of using Curcumin, Chrysamine G have been investigated. A recent study has shown beneficial effects of Curcumin in reducing cognitive defects (10). Chemical structures of compounds investigated in this study are shown in Fig. 1. Nomenclature is according to repeat unit between backbone skeleton. Fig. 2 illustrates conformational reorganization required in Valinomycin to capture K^+^ ions or Zn^2+^ ions and the electrostatic stabilization gained thereafter. It is evident from figure that size of Valinomycin is most suitable for capturing K^+^ ions. Zn^2+^ ions are considerably smaller hence, huge reorganization is required to achieve electrostatic stabilization. Conformational reorganization required plays decisive role in determining ion to be carried by molecule.

**Figure 3:**
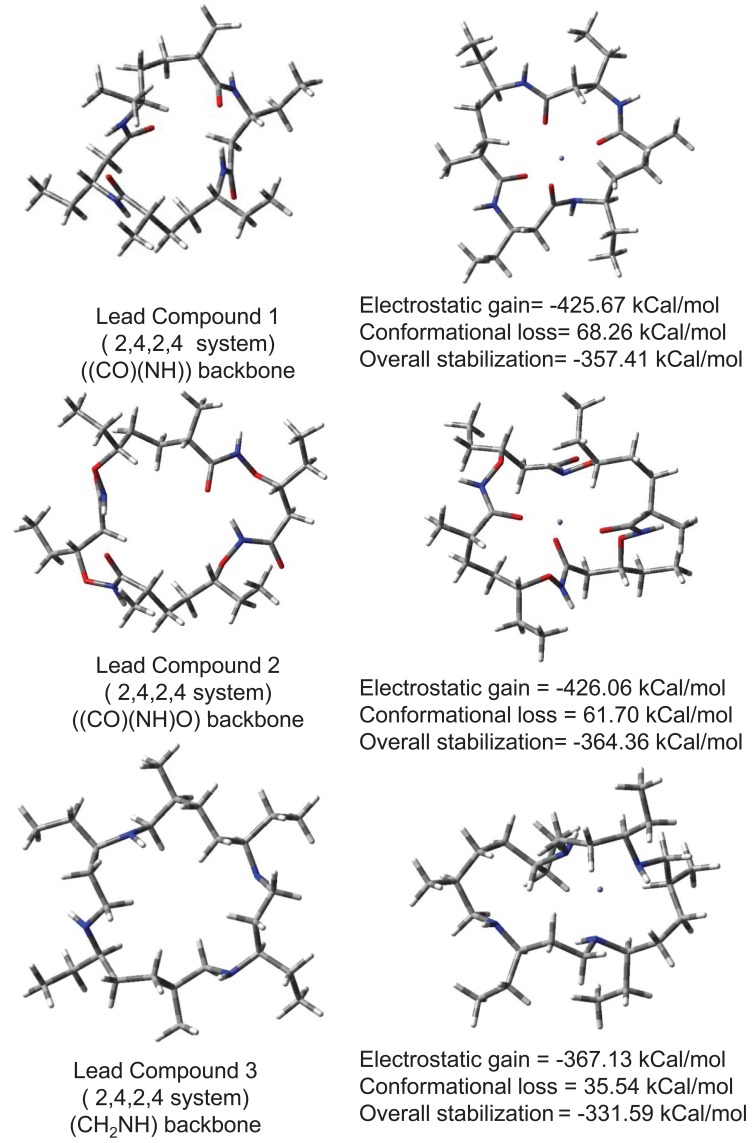
Conformational reorganization in lead compounds 1,2 and 3 to bind Zn^2+^ Ion.

**Figure 4:**
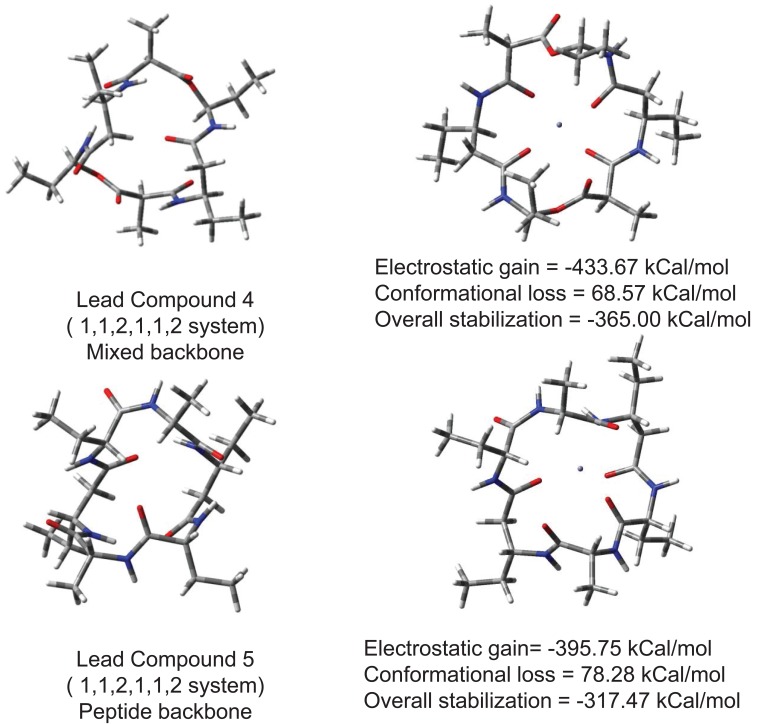
Conformational reorganization in lead compounds 4 and 5 to bind Zn^2+^ ion.

To remove neurotoxic zinc ions from brain (when produced in surplus quantity) a lower molecular weight compound that can easily reorganize and capture Zn^2+^ ions is required. Choice of cyclic ionophore facilitates designing compound that can cross BBB or cell membranes holding a polar ion similar to a carrier protein. Fig. 3 shows conformational reorganization required in lead compounds 1, 2 and 3 for binding Zn^2+^ ion. Lead compound 2 contains a backbone ((CO)(NH)O) slightly more flexible than peptide backbone. Hence lead compound 2 requires slightly lesser energy to reorganize its conformation in presence of zinc ion. Lead compound 3 with CH_2_NH backbone is most facile in reorganizing due to inversion at nitrogens. The molecular weight of compound is also suitable for crossing BBB. Electrostatic stabilization of ion inside ionophore is good enough so that ion would not drift away, and get lost but remain held until flushed out of body. Overall electrostatic stabilization is better with polar backbone. Practical usage of such compounds as drugs however requires low reorganization. Analogous to Valinomycin polarity of backbone was increased in lead compounds 4 and 5 to assess further beneficial effects if any. Conformational reorganization required to capture zinc ions still remains very high. These results are shown in Fig. 4. Increasing polarity of backbone neither increased electrostatic stabilization nor increased conformational flexibility. Efficient reorganization of such peptidomimetic compounds to capture particular ions depends on complex interplay of backbone type, size, polarity; conformational reorganization required in presence of ion and electrostatic stabilization of ion.

Calculated ADME properties of all lead compounds are shown in Table 1. It is clear that lead compounds 2 and 5 are predicted to have very poor oral absorption property. Lead compounds 1 and 3 may have good oral absorption. This study is focussed at designing low molecular weight compound capable of crossing BBB and removing zinc ion toxicity from human brain. Lead compound 3 that is, ((gly-et)-(ala-gly-gly-et))_2_ with CH_2_NH backbone fulfils maximum criteria and is suggested as lead compound for curative/preventive drugs in Alzheimer’s.

**Table 1:** ADME properties of designed lead compounds

Title	Molecular weight	Solvent accessible surface area Unit Å^2^	Number of hydrogen bond donor	Number of hydrogen bond acceptor	Globularity	Hydrophobicity	% Oral absorption	Number of violations of Lipinski’s rule

Lead compound 1	480	742.356	2	8	0.86667	-1.15475	96.6762	0
Lead compound 2	544	807.976	4	16.8	0.839318	-2.06544	37.3143	2
Lead compound 3	424	805.676	4	6	0.817048	-0.6311	88.3827	0
Lead compound 4	512	804.201	0	10	0.81359	-1.76472	51.3151	2
Lead compound 5	510	730.406	1	10	0.860249	-1.5151	27.7251	3

**Figure 5:**
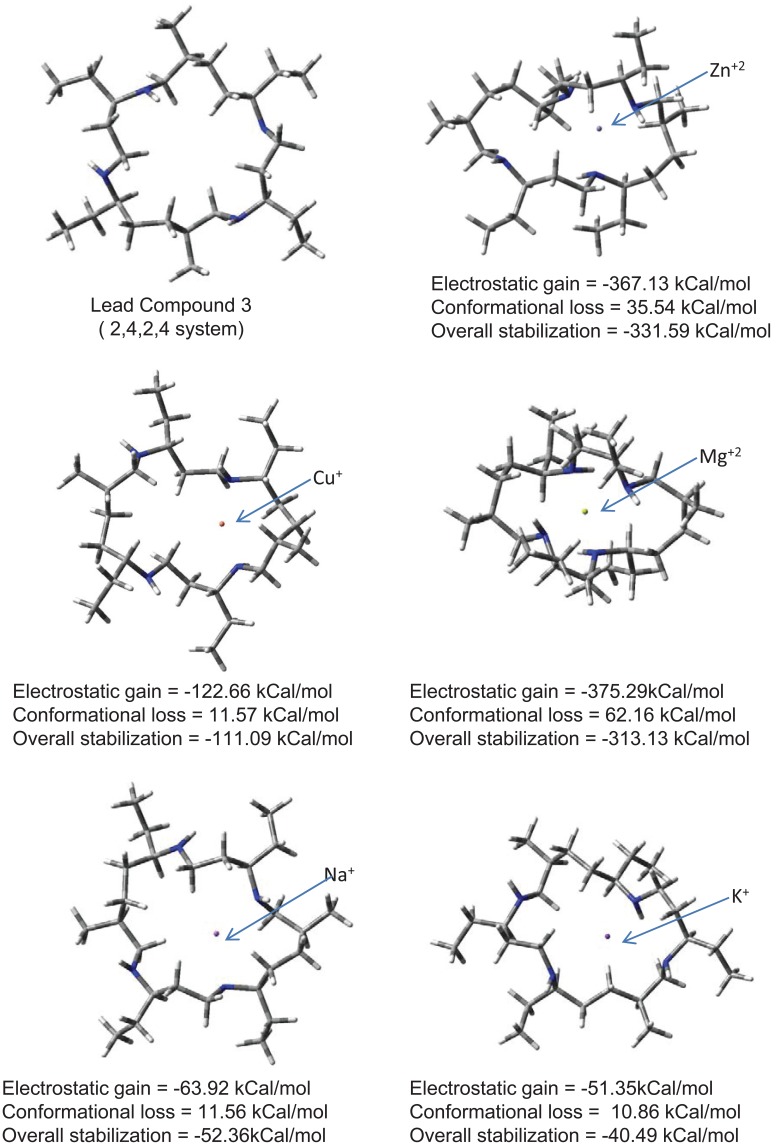
Conformational reorganization in lead compound 3 to bind various metal Ions.

Lead compound chosen for further drug development should be specific for Zn^2+^ ion removal so that it does not remove other vital ions from body creating toxicity and other side effects. Ion specificity issue is addressed in Fig. 5. Capacity of lead compound 3 to capture ions other than Zn^2+^ is shown. Reorganization required to carry Na^+^ and K^+^ ions is low as they are too large to be carried inside ionophore and are therefore carried at the periphery. Table 2 gives a comparison of ionic radii, carriage efficiency of ion by lead compound 3 and reorganization of lead compound required in presence of ion. Results indicate that huge conformational change is required to carry Mg^2+^ ion. Hence, Mg^2+^ ions cannot compete with Zn^2+^ ions for carriage. Cuprous ions can compete with zinc ions for carriage as little reorganization of lead compound is required. However, lead compound would preferentially remove zinc ions as much better overall stabilization is attained with zinc ions If small amount of cuprous ions are removed it would be beneficial for Alzheimer’s patient as presence of cuprous ions in brain leads to enhanced Aß aggregation (22). ((Gly-et)-(ala-gly-gly-et))_2_ system with CH_2_NH backbone is thus suggested as lead compound for reducing neurotoxicity associated with AD.

**Table 2:** Carriage efficiency of various metal ions by lead compound 3

Ion	Ionic radii (pm)	Overall stabilization of ion (Carriage efficiency) (kCal/mol)	Drug reorganization required (kCal/mol)

Mg^+2^	72	-313.13	62.16
Zn^+2^	74	-331.59	35.5
Cu^+^	77	-111.09	11.57
Na^+^	102	-52.36	11.56
K^+^	138	-40.49	10.86

## CONCLUSION

A series of cyclic peptidomimetic ionophores have been studied for their prospective usage as curative/preventive drug for Alzheimer’s. Lead compound 3 containing CH_2_NH peptidomimetic backbone in a 2,4,2,4 cyclic system with gly, ala and ethyl type substituents is suggested as optimum choice with a balance of low conformational reorganization coupled with moderate electrostatic stabilization of zinc ion. Molecular weight of this lead compound is appropriate with hydrophobic groups disposed outwards for facile diffusion through cell membrane and BBB. Calculations also indicate that this compound will selectively remove zinc and copper metal ions from human body.
